# Low-frequency oscillations employ a general coding of the spatio-temporal similarity of dynamic faces

**DOI:** 10.1016/j.neuroimage.2017.06.023

**Published:** 2017-08-15

**Authors:** Nicholas Furl, Michael Lohse, Francesca Pizzorni-Ferrarese

**Affiliations:** a Department of Psychology, Royal Holloway, University of London, Surrey TW20 0EX, United Kingdom; b Cognition and Brain Sciences Unit, Medical Research Council, Cambridge CB2 7EF, United Kingdom; c Department of Physiology, Anatomy, and Genetics, University of Oxford, Oxford OX1 3QX, United Kingdom

**Keywords:** Action perception, Emotional expressions, Face perception, Neural oscillations, Magnetoencephalography, Representational similarity analysis

## Abstract

Brain networks use neural oscillations as information transfer mechanisms. Although the face perception network in occipitotemporal cortex is well-studied, contributions of oscillations to face representation remain an open question. We tested for links between oscillatory responses that encode facial dimensions and the theoretical proposal that faces are encoded in similarity-based “face spaces”. We quantified similarity-based encoding of dynamic faces in magnetoencephalographic sensor-level oscillatory power for identity, expression, physical and perceptual similarity of facial form and motion. Our data show that evoked responses manifest physical and perceptual form similarity that distinguishes facial identities. Low-frequency induced oscillations (< 20 Hz) manifested more general similarity structure, which was not limited to identity, and spanned physical and perceived form and motion. A supplementary fMRI-constrained source reconstruction implicated fusiform gyrus and V5 in this similarity-based representation. These findings introduce a potential link between “face space” encoding and oscillatory network communication, which generates new hypotheses about the potential oscillation-mediated mechanisms that might encode facial dimensions.

## Highlights

• Identity and expression of dynamic faces statistically relate to form perception.
• Only facial expression relates to motion perception.
• Neural rhythms < 20 Hz encode similarity related to form, motion and identity.
• Evoked responses encode mainly facial identity.
• The brain’s oscillatory mechanisms employ similarity-based codes for perception.

## Introduction

Neural oscillations (rhythmic neural firing) are ubiquitous features of the brain and furnish mechanisms contributing to network communication (Engel and Singer, 2001, Salinas and Sejnowski,). Synchronization of membrane potentials enhances coupling between brain regions, allowing them to control information flow and organize specific functional networks (Fries, 2005, Fries, 2009). Hierarchical processing among visual areas may be mediated by oscillatory mechanisms, with forward (bottom-up) and backward (top-down) communication between higher- and lower-level visual areas carried respectively by high- (gamma) and low- (beta) frequency oscillations (Michalareas et al., 2016). These connectivity mechanisms could enable “binding” of visual dimension representations into unitary object percepts (Engel and Singer, 2001). Although these mechanisms have perhaps been best-studied for visual processes in non-human animals, neural oscillations are also a hallmark of visual processing in humans. Low-frequency power modulation is a ubiquitous feature of visual responses measured by electroencephalography (EEG) and magnetoencephalography (MEG). A negatively-deflected alpha/beta (10–30 Hz) response, in particular, putatively indexes visual object encoding (Hanslmayr et al., 2012). Nevertheless, more could be learned about how this low-frequency power deflection gives rise to visual encoding, what information is encoded, and in what format.

An example of a brain network in the human whose communication may be mediated by oscillatory mechanisms is the well-studied network of discrete functional areas in ventral occipital cortex, fusiform gyrus, V5 and superior temporal sulcus (Haxby et al., 2001, Furl et al., 2015) associated with perception of dynamic faces and localized using functional magnetic resonance imaging (fMRI). These face-selective and motion-sensitive areas encode the form and motion information used to recognize faces and their emotional expressions and presumably give rise to oscillatory signals that reflect this encoding and that would be detectable using MEG. For example, spatial locations in static photographs of facial forms useful for expression categorization is reflected in both power and phase of oscillations below 25 Hz (Schyns et al., 2011). Several studies have now also examined dynamic facial movements and illustrated a role for low-frequency oscillations. This frequency range is modulated by motion and form information present in facial video (Muthukumaraswamy et al., 2006, Virji-Babul et al., 2007, Popov et al., 2013, Furl et al., 2014, Güntekin and Başar, 2014, Jabbi et al., 2015, Fox et al., 2016; Symons et al., 2016). These findings suggest that oscillations, especially in low frequencies, may be transmitting the information about form and motion processed in the aforementioned face perception network.

We propose to go beyond these existing studies by investigating the role of neural oscillations in face perception from the standpoint of similarity-based representations. A longstanding theory (Valentine, 1991) of face recognition posits a similarity-based “face space”, where faces are encoded relative to a set of constituent attributes in a multidimensional feature space and evaluated based on their similarity with learned representations. This formulation motivated us to test whether oscillatory power might also reflect representational distances between faces based on their physical and perceptual similarity. Such similarity-based object representations have been discovered using time-domain data from EEG (Kaneshiro et al., 2015), MEG (Cichy et al., 2014), intra-cranial recording (Op de Beeck et al., 2001, Kiani et al., 2007) and fMRI (Haushofer et al., 2008; Drucker and Aguirre, 2009; Proklova et al., 2016) and for static facial attributes such as identities (Vida et al., 2017), configurations (Goesaert and Op de Beeck, 2013) and gaze directions (Carlin et al., 2011). However, these results are limited to time-domain data, and they cannot link stimulus information content with potential neural mechanisms manifested by oscillatory power. Much, therefore, remains to be learned about how oscillations might (or might not) reflect similarities among faces, relative to constituent features in a multidimensional similarity space.

Here, we tested for similarity-based oscillatory responses using representational similarity analysis (RSA) to compare similarity distances between MEG response patterns with similarity values derived from physical and perceptual measures of high-level facial dimensions and categories (Su et al., 2012). To this end, we developed “physical similarity spaces” by extracting configurations of facial form and patterns of facial motion from videos of dynamic facial expressions. We also developed “perceptual similarity spaces”, based on participants’ similarity judgments of facial form and motion. Lastly, “categorical similarity spaces” were based on the between- versus within-category structure for identity and emotional expression. Using these spaces, we were able to behaviorally test for inter-relationships between physical and perceptual measures of facial similarity and whether they contain information about facial identities and emotional expressions. Our main aim, however, was to establish whether any of these similarity spaces was manifested by induced oscillatory MEG responses, as measured at the sensor-level. As a basis for further comparison, we also tested whether time-domain evoked response similarity corresponded to physical perceptual or categorical face spaces. We therefore could determine whether any facial encoding we found for induced responses was also present in evoked signals. Lastly, as a supplemental analysis, we optimized a source reconstruction to localize our sensor space RSA effects within the aforementioned, well-studied face perception network. We acquired fMRI functional localizer data in the same participants as those who underwent behavioral and MEG testing and exploited the superior spatial resolution of fMRI to constrain our source solution. This multimodal dataset of physical data extracted from video, behavioral data, evoked and induced sensor-level MEG responses and fMRI-guided source localizations provided us with a rich set of measures to fully explore several novel tests about representations of facial similarity spaces.

## Methods and materials

### Participants

Twenty participants (> 18 years) were scanned using fMRI. Of these, two did not return for the behavioral experiment, one additional participant did not return for MEG, and behavioral data for one more participant were lost due to technical issues. Analyses proceeded with the sixteen participants who possessed the full complement of data. All participants were right-handed, had normal or corrected-to-normal vision and reported no history of psychiatric or neurological disorder. The local Cambridge, UK ethics committee granted approval.

### fMRI procedures and analysis

Structural scans were obtained to facilitate data registration during MEG source reconstruction. The results of fMRI localizer scans were also used to constrain source solutions to fMRI-defined functional regions of interest (ROIs). fMRI scans were collected using a 3 T Siemens Tim Trio MRI scanner with 32 channel head coil. Functional scans included whole-brain T2*-weighted echo-planar volumes with 64 × 64 matrix and 3 mm^2^ resolution in-plane and 3.75 mm thick axial slices, TR 2 s, TE 30 ms, flip angle 78°. Structural scans were T1-weighted MPRAGE with 1 mm^3^ voxels. The two localizer runs (175 volumes) were separated by runs related to a different experiment on faces, not reported here. The localizer procedures were adapted from Furl et al., 2013, Furl et al., 2015. The experiment was controlled using E-Prime (Psychology Software Tools, Pittsburgh, PA). In each run, participants viewed four types of block, each containing grayscale presentations of a stimulus category: dynamic faces, dynamic objects or static versions of the same faces or objects (taken from the last frame of each video). There were six blocks of each block type per run and block order was pseudo-random. Each block comprised eight presentations of 1375 ms stimuli and a 1 s inter-block interval. Each participant fixated on a white dot overlaid on the center of each presentation and pressed a button-box key with the right index finger when the dot turned red on a pseudo-random one-third of stimulus presentations. Four male and four female facial identities, exhibiting transitions from neutral to disgust, fearful, happy and sad expressions were taken from the Amsterdam Dynamic Face Expression Set (ADFES) (Van der Schalk et al., 2011). Face blocks comprised eight identities and four randomly-selected expressions, with each expression appearing twice. Object blocks included eight objects, previous used in functional localizers (Fox et al., 2009; Furl et al., 2013, Furl et al., 2015). Dynamic object videos included various plants blowing in the wind, a spinning globe, a spinning ceiling fan, a burning flame, operating machinery and a running tap.

fMRI data were preprocessed and analyzed using SPM12 (Wellcome Trust Center for Neuroimaging, London http://www.fil.ion.ucl.ac.uk/spm/) and MATLAB (The Mathworks, Natick, MA, USA). Data were motion-corrected, spatially-normalized to an EPI template in MNI space, and smoothed to 8 mm FWHM. At a first level of analysis, we estimated within-participant effects using an AR(1) corrected general linear model with a 128 ms high pass filter. Four regressors were added by convolving onset times and durations for dynamic faces, static faces, dynamic objects and static objects with a canonical hemodynamic response function. Regressors were also added for head motion parameters. We tested contrasts of the block types at a second level, where a group analysis was conducted to identify locations in MNI space of occipitotemporal areas associated with form and motion representations of dynamic faces (Haxby et al., 2001; Furl et al., 2015). We localized face-selective areas: bilateral occipital face area (OFA), bilateral fusiform face area (FFA) and right superior temporal sulcus (STS) (defined by contrasting face blocks > object blocks) and motion-sensitive areas: right and left V5 (defined by contrasting dynamic blocks > static blocks). For ROI definition, we identified the coordinates of the peaks of clusters observed at *P* < 0.001 uncorrected that achieved family-wise error correction at the voxel level using random-field theory (Brett et al., 2003).

### Behavioral procedures

**Fig. 1:**
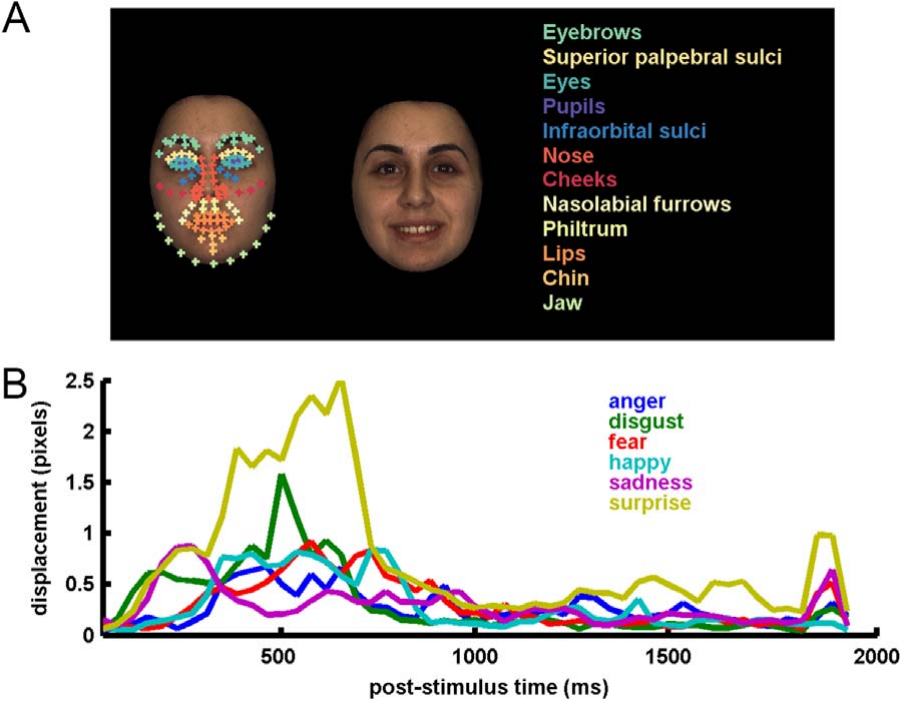
Physical stimulus measures. (A) Examples of neutral (left) and happy (right) video frames. The neutral frame has superimposed on it the landmark locations used to compute configural form and motion pattern measures, color-coded according to facial feature membership. (B) Optic-flow estimates of pixel displacement, averaged for each expression.

The behavioral experiment was conducted using PsychoPy (Peirce, 2009) in a separate testing session either immediately following fMRI or within two weeks. Participants viewed the 630 possible unique pairings of 36 dynamic faces. The 36 faces were taken from the BU-4DFE face set (Yin et al., 2008) and depicted six identities (three female). All videos began with a neutral expression on the first frame (Fig. 1A, left) and then transitioned to the apex of six possible emotional expressions: anger, disgust, fear, happy, sadness and surprise (Fig. 1A) within a 2 s video clip. The timing by which this neutral to emotion movement occurred depended on the individual dynamics associated with each identity and expression (See Fig. 1B for average dynamics for each expression). The two videos on each trial were presented on random sides of the computer screen and looped until the participant responded, so participants could inspect the forms and movements for as long as they needed before making judgments. Participants judged the similarity of each pair by using the mouse to click on a horizontal line on the screen, which represented a continuous similarity scale. Participants judged all pairs twice, once for form and once for motion similarity, in an order counterbalanced over participants. Participants were instructed to differentiate their form and motion judgements based on features, shapes and other information in the video that either were visible across every frame (form) or changed from frame to frame (motion). For form judgments, we instructed participants to evaluate only the forms and shapes visible on every static frame of each video and to ignore anything that changed from frame to frame, including motion. For motion judgments, we instructed participants to evaluate only how faces changed across frames and to ignore information visible across the frames. Although we gave them this guidance for what “form” and “motion” meant, we did not direct their attention to any specific information or features nor did we provide them with specific examples of forms or movements to use. Instead, participants were told that they should decide these for themselves, as we were interested studying their choices. Similarity judgments averaged over participants were used to construct form and motion-based similarity matrices. The form and motion judgment similarity matrices could then be used for RSA to test our hypothesis that MEG oscillatory signals exhibit representations of perceived form and motion.

### Configural form and motion pattern similarity measures

In addition to constructing similarity matrices to characterize subjective, perceived form and motion, we also constructed similarity matrices to characterize more physical, objective measures of form and motion. We then could use RSA to test further hypotheses about the relationship of brain signals with these physical measures of facial form and motion. We extracted physical approximations to form and motion information from the 36 videos used in the behavioral and MEG experiments. One hundred seventy nine image landmarks were detected and tracked over frames using established methods implemented by the Psychomorph software (Chen and Tiddeman, 2010, Yu and Tiddeman, 2010).

**Fig. 2:**
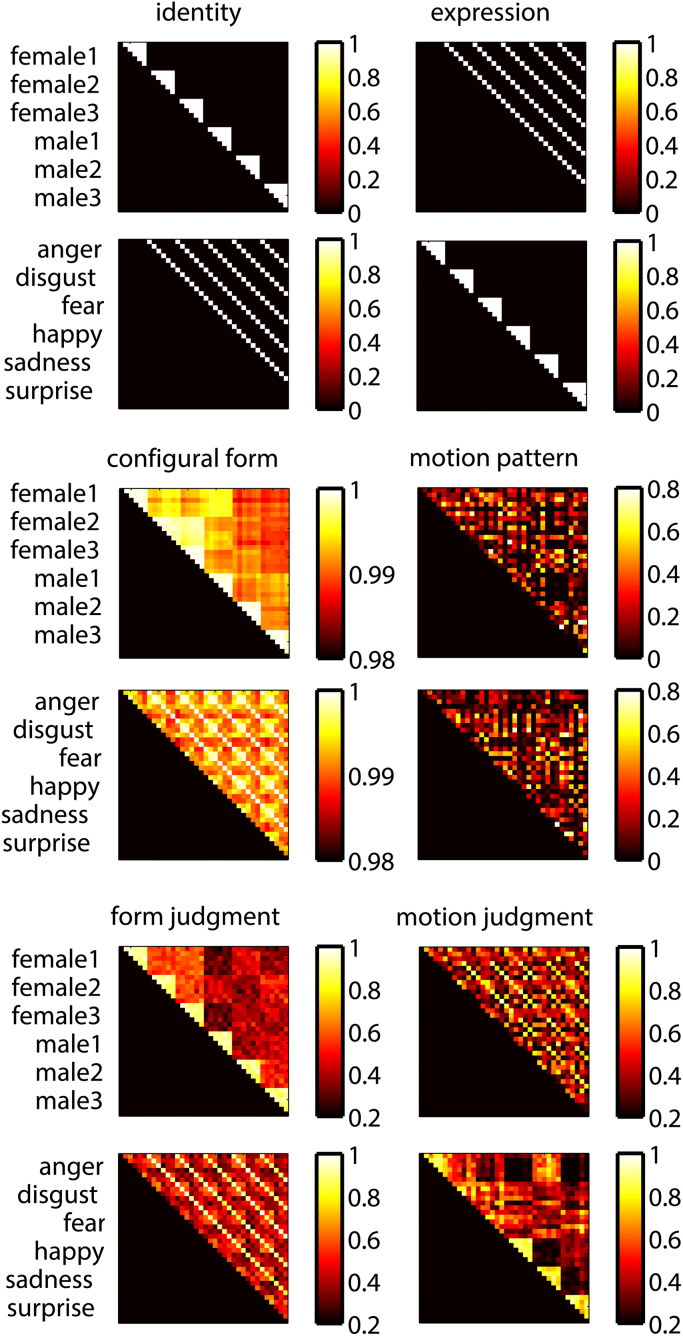
Test similarity matrices. Similarity matrices for physical and perceptual measures of form and motion. The labels on the left side of matrices indicate the positions within the matrices of category members. The matrix rows can be sorted by identities first, then by expressions or by expressions first, then by identities.

We extracted a physical “configural form” measure using landmarks from the first frame of each video, where only a neutral expression was present (Fig. 1A). We computed a “configuration” as the 15,931 element vector of two-dimensional Euclidean pairwise distances between the 179 landmark coordinates. This form measure is “configural” in the sense that it is defined by distances between corresponding points that are defined by high-level facial feature locations, rendering this form representation specific for faces and not computable for non-face objects, which do not have corresponding reference points and therefore no comparable configuration of distances. We populated a configural form similarity matrix (Fig. 2) by taking each pair of videos and computing the Pearson correlation between their landmark configuration vectors.

We also used these landmark positions to approximate a physical measure of the facial motion pattern. To focus our analysis on non-rigid changes in facial muscle position, we selected 141 landmarks within the interior of the face. These landmarks are more likely to be subject to non-rigid expression motion of moveable facial features (Fig. 1A) than exterior landmarks representing head shape. We corrected these landmark positions for rigid, whole head movement by identifying a triangle of three fiducial landmarks on the nose (a structure that can only move with the whole head) and applying an inverse affine transformation to correct the rest of the landmark positions for these fiducial positions. Then, for each video frame, we computed the optic flow (in units of numbers of pixels displaced) for each landmark position, relative to the previous video frame. For computational efficiency, we averaged these optic flow values over selected groups of landmarks associated with facial anatomy. The landmarks included in each feature are color-coded in Fig. 1A and their optic flow values are summarized in Fig. 1B by averaging each expression. Motion typically reaches a visible maximum before 1000 ms, although motion profiles are variable. In a similar fashion to the configural form computation, we found a spatiotemporal motion configuration by computing the 217,470 element vector of Euclidean distances between every pair of optic flow values across the 12 features and 56 video frames. This “motion pattern” captures the three-dimensional optic flow distribution over two spatial position dimensions and the time dimension. As with our configural form measure, we have defined motion as a “pattern” between correspondence points so this high-level representation is face-specific, is not computable for non-face objects, and does not represent the average motion energy or an “overall” motion measure. Similarity matrices were then constructed by taking each pair of videos and computing the Pearson correlation between their motion patterns.

### MEG procedures and analysis

MEG data (306-channel Elekta Neuromag Vectorview system, Stockholm) were sampled continuously at 1 kHz. Participants’ heads were localized within the MEG dewar using five indicator coils. Head shape was characterized by digitizing nasion, left and right preauricular fiducial locations and approximately 80 additional locations evenly-distributed over the scalp. The experiment was controlled using E-Prime. Participants viewed the same 36 videos from the behavioral experiment. Participants viewed all videos eight times in each of four scanning runs resulting in 1152 trials and 32 presentations of each video per participant. Each participant fixated on a centrally-overlaid white dot and pressed a button-box key with the right index finger when it changed red on a random one-third of trials. Videos were sequentially-presented pairs with stimulus onset asynchronies of 3.4 s within each pair of images and 5 s between pairs. Expressions and identities were matched or mismatched over face pairs, for the original intention of measuring repetition suppression. However, repetition suppression effects did not prove robust or significant and are not reported further. We speculate that the lack of repetition suppression may relate to the fleeting, dynamic nature of stimulus information (as repetition effects may be enhanced through prolonged exposure) or to the long stimulus durations and interstimulus intervals we used, which might attenuate repetition effects.

**Fig. 3:**
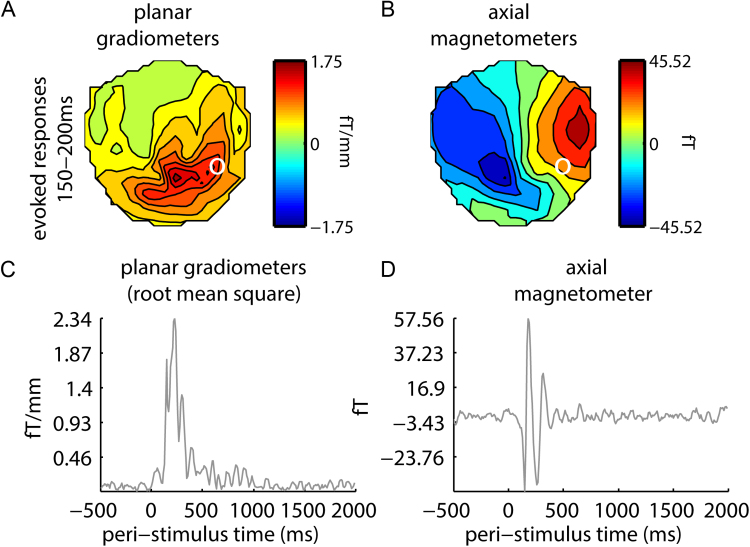
Amplitude components of sensor space evoked responses. Interpolated sensor topographies and contour plots of the evoked response amplitudes of the root mean square of paired planar gradiometers (A) and axial magnetometers (B), averaged between 150–200 ms, a time period selected to capture the M170 component. Also shown are time courses at single sensors located by the white circle in A and B (selected to illustrate the M170) for the root mean square of a pair of planar gradiometers (C) and an axial magnetometer (D).

We used Neuromag Maxfilter (Taulu and Kajola, 2005) to register scalp data, remove artifactual background noise (using a signal separation method) and downsample to 250 Hz. Using SPM12, continuous data were filtered 4–50 Hz, epoched from 500 ms pre-stimulus onset to 2500 post-stimulus onset and downsampled to 100 Hz. Trials were considered artifactual and excluded from analysis if an axial magnetometer signal exceeded 2000 fT or a planar gradiometer signal exceeded 50 ft/mm. For time-domain evoked analysis, epochs (stimulus onset at 0 ms to 500 ms past stimulus offset) were baseline-corrected using the average response −500 to 0 ms (i.e., stimulus onset) and then averaged over trials (Fig. 3). For time-frequency oscillatory responses, we estimated power for 4–50 Hz and −500 to 2500 ms by subjecting epochs to a Morlet wavelet decomposition with factor 7. Epochs in the time-frequency domain were averaged and rescaled by taking, for each frequency, a log-ratio baseline correction with −350 to −100 baseline.

**Fig. 4:**
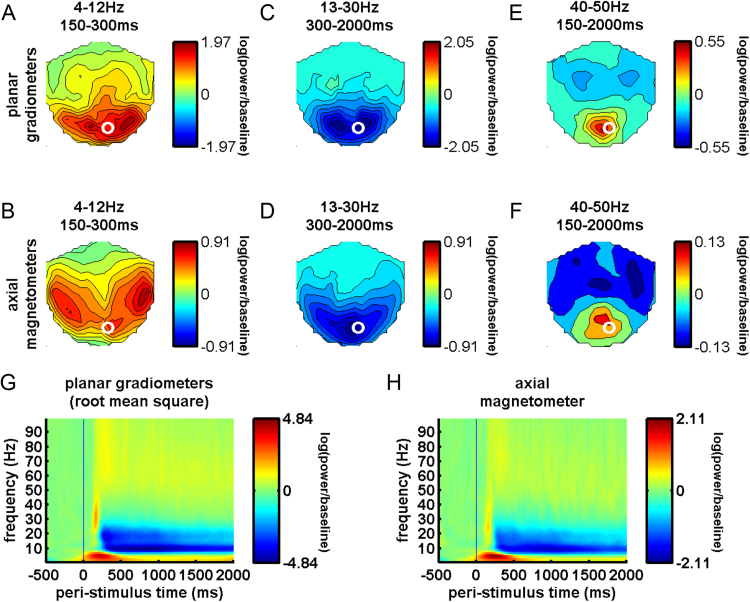
Amplitude components of sensor space oscillatory responses. Interpolated sensor topographies and contour plots of the oscillatory power amplitudes of the root mean square of paired planar gradiometers (top row) and axial magnetometers (middle row), illustrating main oscillatory response components, including: positive occipitotemporal power in theta/alpha range (4–12 Hz), averaged between 150 and 300 ms (A, B); negative occipitotemporal power in the beta range (13–30 Hz), averaged between 300 and 2000 ms (C, D); and positive occipitotemporal gamma band power (40–50 Hz), averaged between 150 and 2000 ms (E, F). Also shown are time frequency representations for the pair of planar gradiometers (G) and the axial magnetometer (H) located by the white circle in A-F. This sensor location was selected to illustrate the time course of all three response components illustrated in A-F.

Our principal predictions that our test similarity matrices would correlate with similarities among MEG responses were tested in sensor space. The MEG sensor-level matrices were based on evoked and induced response similarity. For evoked responses, similarity matrices were constructed at every peri-stimulus time point by taking each pair of videos and computing the Pearson correlation between their patterns of MEG response over all 306 sensors. Because all oscillatory response components manifested overlapping occipitotemporal sensor distributions (e.g., Fig. 4), we could eliminate noisy/irrelevant sensors by selecting sensors where *t*-tests indicated an occipitotemporal response greater than zero at *P* < 1×e^−4^ uncorrected for any frequency or post-stimulus time (56% of sensors). For every peri-stimulus time point and frequency, we constructed a similarity matrix by taking each pair of videos and computing the Pearson correlation between their sensor response patterns.

We hypothesized that low-frequency oscillations would encode facial similarity space dimensions, with a special interest in the negative alpha/beta deflection (as discussed in the Introduction), and so we selected our data, downsampling rate and filters to optimize our analysis for a relatively low frequency range. Nevertheless, we adopted wide enough frequency coverage to include theta, alpha, beta and gamma bands and all the major response components, as shown in Fig. 4. This included even the gamma component, which appears maximal from 40 to 60 Hz and is weaker above 60 Hz. Given a report that similarities among different pictures of faces were related to >80 Hz electrophysiological responses in intra-cranial recordings (Davidesco et al., 2014), we also pursued an exploratory analysis of high gamma (50–100 Hz), using a corresponding 50–100 Hz filter on continuous data downsampled to 200 Hz. We found no significant RSA effects for such high frequencies at *P* < 0.05 using threshold-free cluster enhancement and the same statistical methods described for our 4–50 Hz analysis. We therefore focus our results reporting on analyses optimized for the 4–50 Hz range that includes the traditional time-frequency response features (Fig. 4).

Although we performed our main RSA analysis on sensor data, which is relatively close to the original MEG signals, we also transformed our signals into source space. We examined source reconstructions of both evoked time-domain and time-frequency responses. For the time-frequency analysis, source reconstruction was only aimed at testing where, within the face perception network, our sensor space effects might have arisen, rather than using source reconstruction to perform a parallel test of our predictions with respect to oscillation frequency. Indeed, our sensor space analysis was already framed as a search through frequency space for RSA effects and the result of this search was that 4–20 Hz was identified as the frequencies best expressing RSA effects (Fig. 6). Our source space RSA, in contrast to the sensor-space RSA, was framed as a search through an anatomic region of interest space for RSA effects. Thus, we searched for individual regions of interest expressing RSA effects, averaged over the 4–20 Hz already-known from the sensor-space analysis to best express RSA effects (Fig. 4).

Individual participant cortical meshes with 20,484 vertices (i.e., the “fine” mesh) were prepared in SPM12 by computing a non-linear spatial transformation between each participant's segmented MPRAGE and a template structural MRI in MNI space (Mattout et al., 2007) and applying the inverse transformation to a mesh derived from the template structural MRI. MEG sensor data were coregistered to the transformed mesh using the three (nasion, left, right preauricular) fiducial points for rigid body registration and the manually-defined head shape points for surface matching (Besl and McKay, 1992). Lead fields were computed using a local spheres head model (Huang et al., 1999) and a Bayesian model inversion was carried out using empirical beamforming (EBB; Wipf and Nagarajan, 2009; Belardinelli et al., 2012). To facilitate statistical comparison, we used a group inversion (Litvak and Friston, 2008) restricted to a common set of ROIs, defined from our fMRI localizer runs using the same participants. Our reconstructions were constrained to 10 mm spheres around the fMRI-defined group peak coordinates of right OFA (MNI: 36 −84 −12), left OFA (MNI: −32 −88 −10), right FFA (MNI: 40 −64 −22), left FFA (MNI: −42 −56 −22), right V5 (MNI: 46 −68 −2), left V5 (MNI: −50 −72 4) and right STS (MNI: 62 −46 20). From these reconstructions, we extracted time courses from every vertex (source location) within our ROIs and subjected them to the same evoked and time-frequency analysis procedures as we performed on the sensor-level data. For evoked source responses, the peri-stimulus time courses for each ROI were averaged over epochs. Similarity matrices were then constructed for each time point for each ROI. For time-frequency source responses, we subjected time courses to Morlet wavelet analysis factor = 7 between 4 and 50 Hz and then averaged the resultant time-frequency data over trials. Rather than repeat our search through the frequencies for RSA effects, we used sensor-level RSA to search frequency space for frequencies showing the strongest RSA effects (4–20 Hz, as can be seen in Fig. 6), and then averaged over these frequencies at the source level. We used these averages over low frequencies to populate similarity matrices for each time point for each ROI for use with RSA.

### Representational similarity analyses

We characterized the information content of our perceived and physical form and motion similarity matrices and tested whether they corresponded to MEG response pattern similarity using RSA. RSA indicates shared information content in similarity matrices by detecting correlations between pairs of such matrices and can be performed between any two similarity matrices, whether they represent physical, perceptual, or brain response pattern similarity (Kriegeskorte, Mur and Bandettini, 2008). RSA proceeded by finding Spearman's rank correlations between pairs of matrices (Nili et al., 2014), separately for every participant, taking Fisher's *r* to *z* transformation of these correlations and then testing the significance of the participants’ correlation coefficients at the group level with a one-sample right-sided *t*-test. We predicted a priori that correlations would be positive, as negative relationships between distances were not predicted and, indeed, would not be readily interpretable. For example, it would be surprising and difficult to explain if participants perceived facial forms to be more dissimilar, the more their physical forms or movements were similar.

Before analyzing MEG response similarity, we performed RSAs to test for inter-relations between six “test matrices”: identity and expression categorical structure, configural form and motion pattern physical information (extracted from the videos) and perceived form and motion judgments (Fig. 2). To test for identity and expression category structure, we developed test similarity matrices (Fig. 2) that assigned ones (maximal similarity) to within identity/expression face pairs and zeros (minimal similarity) to between identity/expression face pairs. Note that our identity similarity matrix tests for similarity structures that distinguish between identities, despite changes in six different expressions, and so is designed to test for expression-invariant identity representations. Likewise our expression similarity matrix tests for similarity structures that distinguish between expressions, despite changes in six different identities, and so is designed to test for identity-invariant expression representations.

Our planned comparisons tested whether: (1) form and motion judgment matrices are positively correlated, (2) form and motion judgment matrices positively correlate with identity and expression matrices, (3) the two physical matrices (configural form and motion pattern) positively correlate with identity and expression matrices, (4) The two physical matrices positively correlate with their corresponding perceptual (form and motion judgment) matrices.

We implemented RSA to statistically test for relationships between (a) the six test matrices (Fig. 2) and (b) MEG evoked response and time-frequency power similarity at sensor-level and source-level. We were also interested in determining whether form and motion representations were structured according to the forms or motions of identity or expression categories, or whether form and motion were represented more generally. Thus, we ascertained whether form and motion judgments uniquely contributed to the MEG signal, after variability due to identity and expression matrices had been removed using Spearman partial correlations.

To increase the signal to noise ratio, based on the matched-filter theorem, we provided mild smoothing to the raw RSA correlations, prior to statistical testing. For evoked timecourses, we applied a low-pass Butterworth filter (< 10 Hz) and for the time/frequency maps, we used Gaussian filtering (4 Hz, 20 ms FWHM). This smoothing step is commonly used as part of univariate analysis, where smoothing/filter kernel choice should reflect, in part, potential inter-participant variability, in order to optimize overlap of the same effect exhibited in different participants. We chose our kernel sizes to be small and conservative, compared to the common uses of such applications to evoked or time-frequency data (Kilner et al., 2005, Kilner and Friston, 2010, Litvak et al., 2011, Perry and Singh, 2014). The resultant data were then submitted to mass-univariate one-sample *t*-tests (right-sided, as all relationships are between similarity distances, and so are expected to be positive). Threshold-free cluster enhancement (TFCE), combined with permutation testing (10,000 iterations), as implemented in the CosMoMVPA toolbox (Smith and Nichols, 2009, Oosterhof et al., 2016) was used for multiple comparison correction for the number of time points (for sensor-level evoked responses and for source-space ROIs) and the maps of time points and frequencies (for sensor-level oscillatory power).

## Results

### Analysis of test similarity matrices

Fig. 2 shows the six test similarity matrices representing identity and expression categories, configural form, motion pattern and perceptual judgments about form and motion. The rows of the similarity matrices can be sorted by identities and then by expressions (first, third and fifth rows, Fig. 2), such that greater within-identity similarity (compared to between-identity similarity) appears as half-triangles near the diagonal, where same-identity pairs group together (first row, Fig. 2). This identity-based categorical structure is visible for configural and perceived form. When the test matrices are sorted, instead, by expression and then by identity (second, fourth and sixth rows, Fig. 2), expression categorical structure appears as half-triangles near the diagonal. This pattern is visible for motion judgments, although some categories (disgust, fear and anger) appear more confusable than others (happy), consistent with previous findings (Furl et al., 2013).

We used Spearman rank correlations (RSA) to formally quantify shared information in the test similarity matrices. First, we found that form and motion judgment matrices shared information *r* = 0.22, *P* < 0.001. Second, we tested for any inter-relationships form and motion judgments might have with identity and expression categorical structures. Identity was associated with form *r* = 0.61, *P* < 0.0001, but not motion judgments *r* = −0.03, *P* = 0.472. Expression was associated with both form *r* = 0.22, *P* < 0.001 and motion judgments *r* = 0.54, *P* < 0.0001. Third, we tested for associations of configural form and motion pattern with identity and/or expression. Identity was associated with configural form *r* = 0.60, *P* < 0.0001 and motion pattern *r* = 0.09, *P* = 0.02. However, expression was associated with neither configural form *r* = −0.09, *P* = 0.99 nor motion pattern *r* = 0.04, *P* = 0.303. Fourth, we tested for associations of configural form and motion pattern with form and motion judgment. We found that configural form related to form *r* = 0.49, *P* < 0.0001 but not motion judgments *r* = −0.009, *P* = 0.825. Motion pattern was related to both form *r* = 0.19, *P* < 0.001 and motion judgments *r* = 0.11, *P* = 0.005. Although we used planned comparisons, the same correlations just reported remained significant after a more conservative Bonferroni correction for the 13 comparisons (critical *P* = 0.008), with the exception of the relationship between motion pattern and identity.

To summarize, Identity, along with the physical and perceptual measures of form composed an inter-correlated group of measures. While expression was also related to form perception, expression further composed a part of a group of inter-correlated measures that included perceived motion perception and (physical) motion pattern.

### Analysis of MEG sensor data

Fig. 3 shows main MEG response amplitude components in peri-stimulus time. We observed an M170 response with its typical latency and response distribution over sensors. A transient increase in theta/alpha power (4–12 Hz) appeared coincident with the M170. This was followed by a negative deflection in the alpha/beta range (8–25 Hz) which was sustained for the remainder of the 2 s epoch, consistent with previous reports (Hanslmayr et al., 2012, Furl et al., 2014). A gamma power component appeared coincident to the M170 and initially broadband. However, this component was sustained throughout the remainder of the epoch, where it was centered on 40–60 Hz. To demonstrate that our selected range for RSA (4–50 Hz) overlaps with this gamma power component, the sensor maps showing positive occipital gamma field power from 40 to 50 Hz are shown in Fig. 3E and F. All three time-frequency response components showed power distributed over posterior occipital and temporal sensors. For RSA, we were interested in testing whether categorical, physical and perceptual similarity was exhibited in MEG responses concomitant with these univariate response amplitude components.

We tested whether similarity among MEG sensor patterns correlated with the six test similarity matrices using Spearman rank correlations. This analysis allowed us to test our principal hypothesis that oscillations, especially in low frequencies, show similarity patterns that match those of physical and perceived facial form and motion and similarities that distinguish identities (across changes in expression) and expression (across changes in identity). The correlations were numerically small (maximum 0.12) but statistically significant and comparable to previous reports including (as examples) split-half Spearman correlations of object recognition similarity fMRI data (Walther et al., 2016) and correlations between facial identity-based similarities in MEG responses (Vida et al., 2017).

**Fig. 5:**
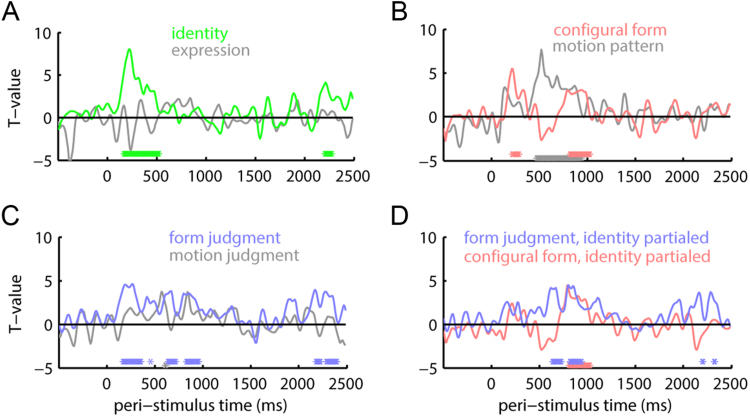
Representation similarity analysis of sensor-space evoked responses. *T*-values for Spearman correlations between sensor-level evoked response pattern similarity and test similarity matrices representing: (A) categorical structure of identity (green) and expression (gray); (B) configural form (pink) and motion pattern (gray); (C) form (blue) and motion (gray) judgments; (D) configural form (pink) and form (blue) judgments, each controlling for identity categorical structure using partial correlation. Shaded areas represent standard errors of the mean. Asterisks at bottoms of graphs indicate time points with Spearman correlations significantly greater than zero at *P* < 0.05 after threshold-free cluster enhancement correction for the number of time points. Asterisks are color-coded by test similarity matrix.

For time-domain evoked responses, we tested our test similarity matrices (Fig. 2) against MEG sensor pattern similarity separately for each time point (Fig. 5) and found that information about identity, configural form and form judgment was exhibited by evoked responses coincident with the M170, peaking circa 150–200 ms, and decaying until before 500 ms. Controlling for identity categorical structure using partial correlation eliminated the relationship between evoked responses and form judgment and rendered any residual relationship with configural form at this early time non-significant. Controlling for expression did not alter any results. At a later time (~900 ms), configural form and form judgment showed a weaker relationship with evoked responses. However, at this time, there was no concomitant relationship with identity and partialing out identity or expression matrices did not alter the relationships between physical and perceived form. Thus, at this later time, a small amount of form information was represented in evoked responses, but not enough to distinguish different identities or expressions. At an even later time period, 250 ms after stimulus offset, evoked responses exhibited representations of identity and form judgment, but did not show a relationship with configural form. Although evoked responses related to motion pattern similarity between 500 and 1000 ms, they showed no relationship with motion judgment at any time. Thus, evoked responses can signal some motion information, but none that factors into perceived motion similarity or expression.

**Fig. 6:**
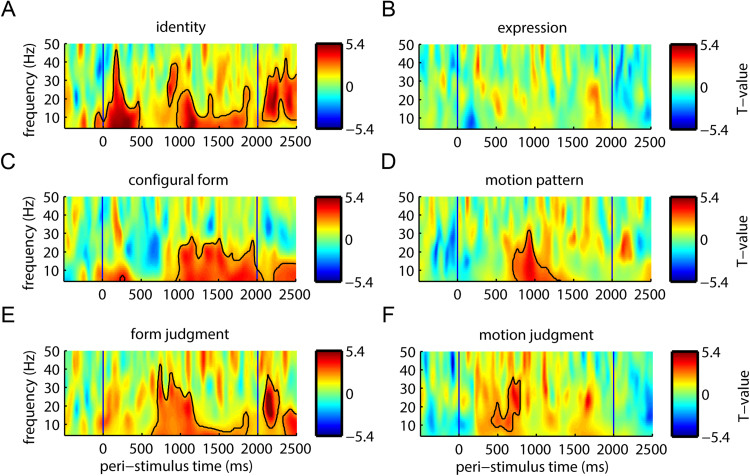
Representation similarity analysis of sensor-space oscillatory responses. *T*-values for Spearman correlations between sensor-level oscillatory power pattern similarity and test similarity matrices representing: (A) identity and (B) expression categorical structure; (C) configural form; (D) motion pattern; (E) form judgments; (F) motion judgments. Black contour lines outline clusters of time-frequency points that are significant at *P* < 0.05 using threshold-free cluster enhancement multiple comparison correction.

Time-frequency domain oscillatory power at the sensor-level revealed positive statistical relationships between low-frequency response pattern similarity and every test similarity matrix (Fig. 6), except for expression. We report *P*-values for the maximal (peak) effect found within the stimulus duration. The identity matrix showed an early effect ~200 ms (peak, *P* = 0.001, corrected), a later effect that extended from before 1000 ms nearly to stimulus offset (2000 ms) and a final effect ~250 ms post-stimulus offset. Configural form, like identity, exhibited an early (but much smaller) relationship with ~5 Hz responses, followed by a larger 4–30 Hz effect extending between 1000 ms (peak, *P* = 0.001, corrected) and stimulus offset, and a final effect 250–500 ms post-stimulus offset. Motion pattern related to 4–30 Hz responses at around 900–1000 ms (peak, *P* = 0.002, corrected). Perceived form related to 4–40 Hz responses at 550–1200 ms (peak, *P* = 0.006, corrected) and also showed later effects in the post-stimulus offset period for 10–30 Hz responses at 250 ms post-stimulus offset and 4–20 Hz responses at 400–500 ms post-stimulus offset. Perceived motion related to 6–30 Hz responses at 400–800 ms (peak *P* = 0.02, corrected).

To summarize the results for oscillatory responses, several test similarity matrices matched the similarity structure of low-frequency oscillations either around 200 ms, or later, between 500 and 1500 ms. At this later time, several measures were roughly aligned in time, often directly overlapping. These measures included identity (900–1900 ms), configural form (900–2000 ms), form judgment (600–1500 ms), motion pattern (600–1200 ms) and motion judgment (500–800 ms). Although our measurements of these timings are unlikely to be exact (due to measurement noise, variable effect sizes, inter-participant variability and variability in stimulus motion timing), it seems, collectively, that they might show convergent results within the period between 500 and 1200 ms. It is thus possible that some of these oscillatory effects reflect a broad, general encoding of form and motion information, that forms the basis for behavioral perceptual reports and contains form information that, at least, can distinguish between different identity categories.

Although there appears to be enough information about form available during this time period to distinguish identities (across changes in expression), these responses may contain more information about form and motion than is needed to differentiate categories like identity or expression. To assess this possibility, we re-tested the relationships between MEG oscillatory responses and test similarity matrices, but partialing out identity or expression in cases when they were collinear. The relationship between MEG time-frequency responses and configural form remained significant after controlling for identity (peak *P* = 0.02, corrected). Relationships with motion pattern were also significant after controlling for identity (peak *P* = 0.004, corrected) or expression (peak *P* = 0.004, corrected). The relationship between MEG time-frequency responses and form judgment remained significant when using partial correlations that controlled for variation in identity- (peak *P* = 0.02, corrected) or expression-based similarity (peak *P* < 0.0001, corrected). The relationship between MEG time-frequency responses and motion judgment remained significant after controlling for expression (peak *P* = 0.02, corrected).

### Analysis of MEG source reconstructions

Our source reconstructions were a supplementary analysis that aimed to identify brain regions from within the face perception network that give rise to the effects that we observed in sensor space. We localized evoked signal and also localized induced signal, averaged within frequencies that we observed in sensor space (Fig. 6) to exhibit RSA results (4–20 Hz). We projected these sensor data onto vertices that correspond to anatomical locations on a cortical mesh. We then analyzed similarity among the patterns of activation distributed over the vertices found within each fMRI-defined brain area.

**Fig. 7:**
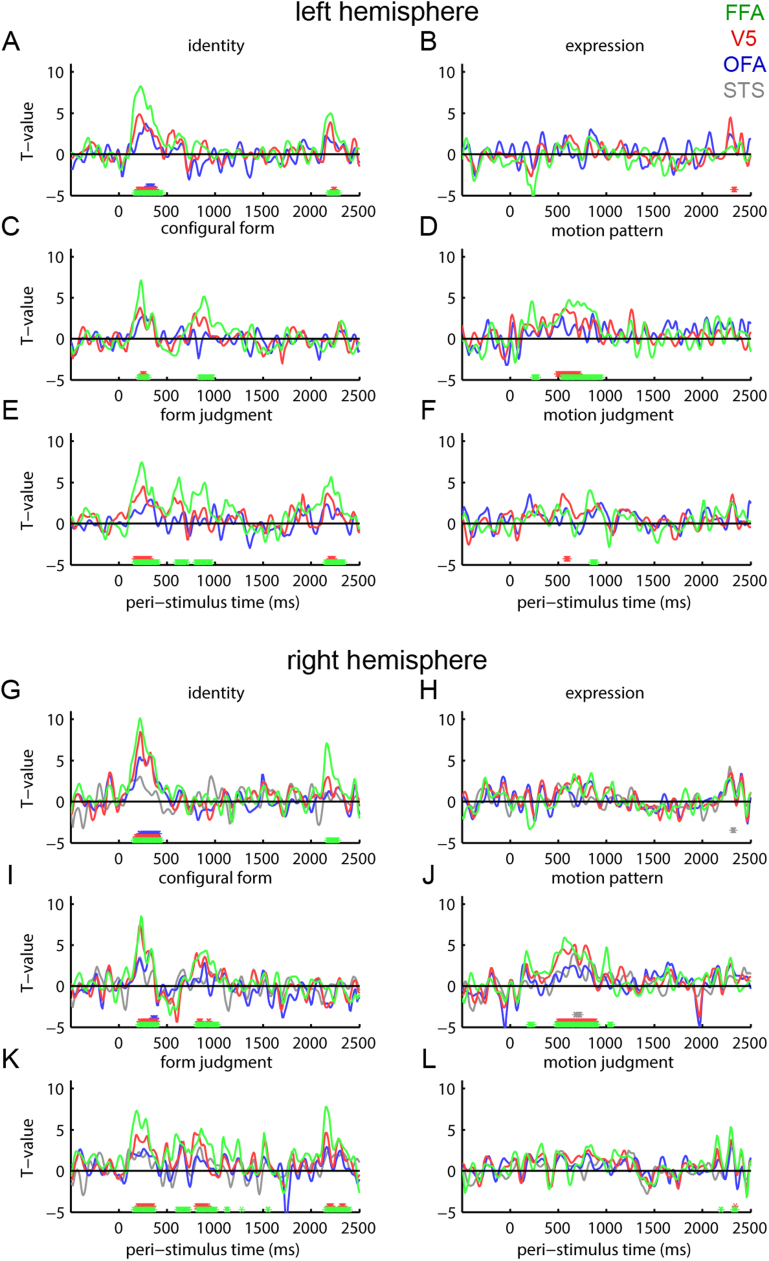
Representation similarity analysis of source-space evoked responses. *T*-values for Spearman correlations between source-level evoked response similarity in fMRI-defined functional regions of interest and test similarity matrices. Lines are color coded by region of interest. Asterisks at bottoms of graphs indicate time points with Spearman correlations significantly greater than zero at *P* < 0.05, threshold-free cluster enhancement corrected. Asterisks are color-coded by region of interest. Panels A-F are left hemisphere data extracted from OFA (blue), V5 (red) and FFA (green). Panels G-L are right hemisphere data extracted from STS (gray), OFA (blue), V5 (red) and FFA (green). Test similarity matrices include (A, G) identity and (B, H) expression categorical structure; (C, I) configural form; (D, J) motion pattern; (E, K) form judgments; (F, L) motion judgments.

For time-domain evoked responses, we extracted these patterns and compared their similarity to our test similarity matrices at every post-stimulus time point. We localized in source space (Fig. 7) the evoked results that we observed in sensor space (Fig. 5) to bilateral FFA and right V5. This activity was related to both early (~200 ms) effects of identity, configural form and form judgment and later effects of configural form, form judgment and motion pattern. Left V5 also contributed to effects in the early time period and to motion pattern. Bilateral OFA also contributed to identity in the early time period. Other findings were small and/or transient.

**Fig. 8:**
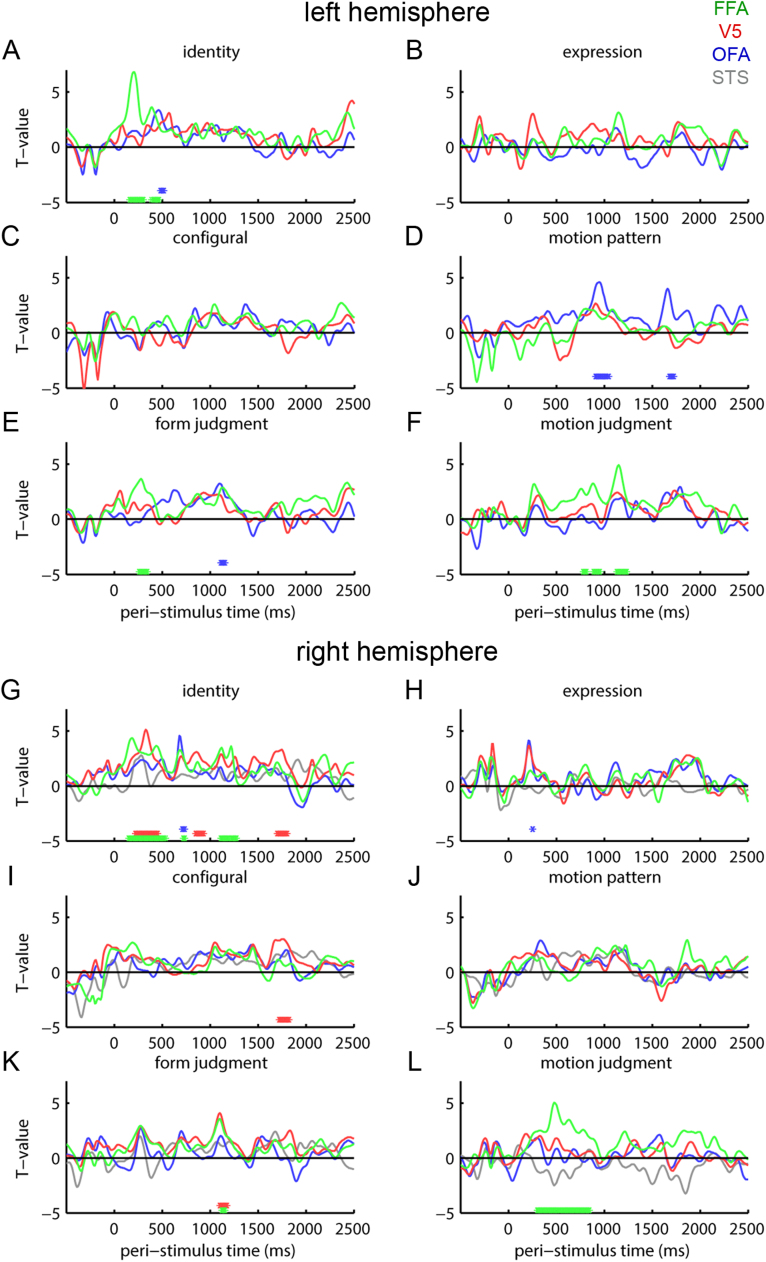
Representation similarity analysis of source-space oscillatory responses. *T*-values for Spearman correlations between source-level oscillatory response (averaged 4–20 Hz) similarity in fMRI-defined functional regions of interest and test similarity matrices. *T*-values for Spearman correlations between source-level evoked response similarity in fMRI-defined functional regions of interest and test similarity matrices. Lines are color coded by region of interest. Asterisks at bottoms of graphs indicate time points with Spearman correlations significantly greater than zero at *P* < 0.05, threshold-free cluster enhancement corrected. Asterisks are color-coded by region of interest. Panels A-F are left hemisphere data extracted from OFA (blue), V5 (red) and FFA (green). Panels G-L are right hemisphere data extracted from STS (gray), OFA (blue), V5 (red) and FFA (green). Test similarity matrices include (A, G) identity and (B, H) expression categorical structure; (C, I) configural form; (D, J) motion pattern; (E, K) form judgments; (F, L) motion judgments.

For time-frequency oscillatory source-level responses (Fig. 8), bilateral FFA was related to the sensor-level RSA effects on identity, form judgments and motion judgments. Right V5 contributed to effects of identity, configural form and form judgments. Left OFA contributed to the RSA motion pattern effects. Other findings were small and/or transient. The configural form RSA effects that we observed at sensor level were not as well-detected in source responses.

## Discussion

### Similarity spaces manifested in behavior and MEG signals

We identified a number of new findings with respect to the structure of similarity space-based encoding in MEG signals, including similarity spaces associated with several physical and perceptual dimensions of dynamic facial video and facial identity. Our results revealed relationships between similarity representations of (1) physical information extracted from facial video, (2) behavioral-reports of perceived form and motion and (3) facial identity and emotional expression.

Physical and perceived form information was associated with identity and, to a lesser degree, expression. In contrast, we found inter-relations among expression and physical and perceived motion. These behavioral RSA findings underscore the differences in physical and perceptual information that support identity and expression recognition. Indeed, our findings are consistent with existing models of identity and expression recognition, which predict that identity and expression perception is based on differing degrees of form and motion information (Haxby et al., 2001, Calder and Young, 2005).

We also applied this same similarity-space technique to identify similarity space dimensions (form, motion) and their associated categorical perceptions (identity, expression) that are represented in MEG activity measures (evoked versus induced oscillatory). We tested this primary hypothesis in sensor space targeting a frequency range that captures three well-known univariate components of MEG responses (alpha, beta, gamma). Identity and physical (configural) form encoding was associated with early (150–250 ms) evoked and induced response components, with stronger physical and perceptual form effects for evoked responses. Later (500–1200 ms), oscillatory responses (<20 Hz), but not evoked responses, showed encoding of several physical and perceptual measures, alongside identity encoding. Because these responses were likely to arise from the oft-reported network of face-selective and motion-sensitive brain areas (that we also measured using fMRI in our participant sample), we computed source reconstructions optimized to test for the locations of our sensor-space findings within these areas. We found evidence primarily for FFA and V5 in encoding physical and perceptual dimensions.

In summary, we offer novel findings with respect to similarity-based, spatiotemporal representations of dynamic faces, which suggest that there are distinct roles for evoked and induced responses in encoding form and motion dimensions, including those that might support identity recognition. Given that neural oscillations manifest fundamental neural communication mechanisms (Fries et al., 2005), our findings provide a new step toward a mechanistic understanding of how the face perception network communicates and gives rise to behavior.

### Measuring dimensions of dynamic faces with physical measures

We used subjective judgments as behavioral measures of perception of the dimensions of facial similarity spaces. However, participants were instructed to choose the most relevant facial information for similarity. The facial information chosen is not obvious to researchers and so we extracted physical measures of form and motion to assist interpretation. Jabbi et al. (2015) successfully used a similar strategy to show that beta power amplitude tracks a physical quantification of facial expression motion. Here, we similarly quantified motion from facial video, but using a motion pattern measure together with RSA to relate this physical measure with both perception and MEG responses. We found correlated similarity between physical and perceived motion, even though our motion pattern measure nevertheless lacked the expression categorical structure that was evident for perceived motion. There is room for further development of this novel approach that might in future better capture movements discriminative of expression categories.

Physical and perceived measures were correlated for form also and both measures exhibited greater between-identity similarity than within-identity similarity. In contrast, neither physical nor perceived motion showed a relationship with identity. Nevertheless, some variation in motion pattern influenced form perception. Facial morphology might influence both a face's invariant static appearance while constraining the movements of the face and so could influence static form, motion and “structure from motion” perception.

### Representations revealed by evoked versus induced responses

In addition to using behavioral RSA to understand the perceptual dimensions of similarity space representations, we also tested for representation of these dimensions in sensor-space evoked time domain and induced oscillatory responses (Fig. 5, Fig. 6). In a relatively early time period, these two measures showed convergent findings, related to identity and configural form encoding. These RSA effects may be related to the M170, or an MEG equivalent of N250. The characteristics of this effect conform to proposals that facial identity information becomes available and is reflected in evoked potentials near to this time period (Schweinberger and Burton, 2003). At least for evoked responses, information during this time period seemed limited to form information organized by identity. Effects relating evoked responses to physical and perceived form measures became non-significant after partialing out the identity matrix.

The identity-specific similarity information encoded during this early time period by evoked responses and low-frequency oscillations must include high-level visual information. Our identity matrix considers similarities among identities across changes in expressions and so the identity code is likely to be expression-invariant. Although a previous study examined identity-based similarities among electrocorticographic gamma responses (Davidesco et al., 2014), invariance across different static photographs pictures of the same identity was not reported. Our evoked response results more closely resemble those of Vida et al. (2017), who show invariant identity decoding for evoked responses to static photographs in the same early time period, although they did not analyze time-frequency representations or dynamic faces. We also show evoked responses and < 20 Hz similarity spaces that manifest structures correlated with our physical configural form measure. This measure captures high-level form information, as it is based on demarcations of shapes, and is computed based on distances that span the face between landmarks. This measure is not derived from local image information and could not be represented by neural populations with small receptive fields, such as in retinotopic cortex.

At later time periods (500–1200 ms), evoked time domain (Fig. 5) and induced oscillatory responses (Fig. 6) were more divergent. While evoked responses no longer encoded identity, time-frequency power (<20 Hz) gave rise to similarity spaces that corresponded to identity as well as physical and perceived form and motion. Although the responses did not all share identical timing, they were approximately coincident and might therefore represent a common representational similarity space. Unlike the early evoked response, these later oscillatory responses reflected both identity-dependent and identity-independent representations of form, as the relationships with both physical and perceived form matrices were robust to partialing out the identity matrix. Low-frequency representations therefore might manifest a general similarity space related to several facial dimensions, with a subset of the information capable of distinguishing identities. In any case, measurement of induced responses were necessary to reveal both motion- and form-based representations that were are not readily measureable in evoked time-domain signals. Thus, our results introduce an important methodological caution, as many EEG and MEG studies limit their analysis to evoked responses.

This finding of general dimensional coding, beyond identity categorical structure complements previous fMRI research. While facial identities are decodable from fMRI response patterns (Anzellotti and Caramazza, 2014), neural response patterns can represent facial information that goes beyond discrete identity membership, including quantitative similarity along perceived dimensions of faces (Carlin et al., 2011, Goesaert and Op de Beeck, 2013, Stolier and Freeman, 2016). For example, Sormaz et al. (2016) shows that physical facial landmark configurations (similar to our configural form measure) and perceptual similarity judgments between static facial expression images correlate with fMRI response similarity even after expression categorical structure is eliminated from the data. This type of this broader, more generalized similarity-based coding (as opposed to coding limited to discrete categories) may be prevalent. Occipitotemporal fMRI responses encode the constituent dimensions of diverse visual stimuli from objects to animals (Edelman et al., 1998, Haushofer et al., 2008, Proklova et al., 2016) and object similarity can be detected in MEG time-domain patterns (Cichy et al., 2014). However, time-domain MEG and fMRI cannot speak directly to whether *oscillatory* neural mechanisms mediate representations capable of producing similarity relationships. Our findings introduce the possibility that neural oscillations may contribute to general coding of stimulus dimensions for dynamic faces and other visual stimulus domains.

One surprising finding from our sensor-space RSA was that no relationships were found between MEG signals and facial expressions. When the participants’ task was to judge perceived form and motion, their judgments distinguished expression categories. In contrast, MEG signals while viewing faces (but not making judgments) did not distinguish emotional expressions. This is not likely due to insufficient power, as our data provided sufficient power to detect relationships between MEG similarities and a number of other types of information, including identity. Few results of successful decoding of dynamic facial expressions from MEG or EEG sensor data are reported to date (but see Tsuchiya et al., 2008, for decoding of morphed emotion transitions from electrocorticography) and so we can only speculate about this post hoc. However, there are a number of mostly non-mutually exclusive possibilities that will need to be considered in future attempts. (1) Facial form information, used for recognizing identity, is available immediately upon stimulus onset. Thus, the timecourse of form processing and identity recognition may be similar across stimuli. However, expressions elapse at variable rates and so transient expression recognitions may not have overlapped sufficiently across participants to yield effects. (2) There is typically substantial variability in the recognizability of different expressions (e.g., happy faces are well-recognized while fearful expressions are not). Fig. 2 shows, for example, that perceived motion does not predict every expression category equally. This may reduce our power for detecting expressions matrices defined for all expressions equally (Fig. 2). (3) Facial expressions may not be all processed simultaneously by a single cortical system (Calder and Young, 2005). Several MEG signals, occurring at different times and from different sources may contribute to expression recognition. Some sources, like the amygdala, may not be as accessible to MEG as others. (4) Our expression matrix required that expressions be distinguished across identities. However, representations accessible to the MEG may be identity-specific. (5) Participants were not instructed to respond or attend to expression or the face, but responded to fixation point color changes. Expression effects might manifest when they are task-relevant.

### Do oscillations mediate face space representations?

What causes brain signals to exhibit these similarity-based relationships? Intracranial recording studies in humans (Op de Beeck et al., 2001) and macaque monkeys (Kiani et al., 2007) show that population coding gives rise to distributed response patterns that reflect similarity relationships among stimulus attributes. Population codes, defined over neurons sensitive to facial form or motion dimensions, also accords with face space theory. This popular theory of face representation (Valentine, 1991) suggests that faces are represented as vectors in a multidimensional similarity-based feature space. Our multivariate RSA results show that low-frequency neural oscillatory response patterns can index a type of similarity-based coding and should serve as candidates for future study on the dimensions of face space representations.

This potential link between face space dimensions and oscillations introduces a new mechanistic perspective on face perception that can be described in terms of network function (Fries, 2005). Empirical evidence and theoretical modeling raise the possibility that low-frequency oscillations in the visual system arise from backward hierarchical connectivity (Bastos et al., 2012, Michalareas et al., 2016). If so, then our data suggest a new hypothesis that top-down processing (i.e., backward connectivity) is key for instantiating similarity space representations. Although hypotheses of such a mechanistic nature remain speculative now, the finding that multidimensional similarity space representations might rely on low-frequency channels that are not necessarily in phase with stimulus presentation (induced responses) introduces novel mechanistic neuroscientific hypotheses that are testable.

### Relationship to oscillatory response amplitude components

To date, links between visual perception and oscillations have relied predominantly on univariate analysis of response amplitudes, rather than the multivariate similarity space approach we employed here. An oft-reported (Hanslmayr et al., 2012) negative deflection in the alpha/beta range (8–12 Hz), arising 250–300 ms after visual stimulation (Fig. 4) appears concomitant with many of our findings. This deflection may be related to the ongoing coding that we were able to detect using RSA. Consistent with our findings, the overall magnitude of alpha/beta power response in previous studies has been modulated by dynamic expression movements (Popov et al., 2013, Jabbi et al., 2015) and speech movements (Muthukumaraswamy et al., 2006), although our study is the first to examine response pattern relationships among individual faces for this frequency range. The Mu rhythm is another negative deflection in a similar frequency range that has been well-studied in the context of body actions (Fox et al., 2016). The mu rhythm might represent forms and motions of bodies, just as low frequency codes represent this information for dynamic faces. However, further studies using RSA (as we used here) and multivariate decoding are needed to go beyond univariate response modulations and to measure the similarity structure of individual stimuli such as bodies.

In addition to these low-frequency modulations, univariate analysis of gamma power (> 30 Hz) typically shows a sustained response component around 40 Hz in response to visual stimuli (Uhlhaas et al., 2011). In MEG responses to static facial photographs (Gao et al., 2013, Uono et al., 2017), this component can also be centered around 40 Hz, although it can be faster (Dobel et al., 2011, Perry and Singh, 2014). This gamma power component was also present for dynamic facial videos in our data, observed between 40 and 60 Hz (Fig. 4). Although this component should have been detectable within our 4–50 Hz sensor-space RSA window, we found limited evidence for similarity-based encoding above about 20 Hz. Several studies examining oscillations with direct cerebral electrical recordings have reported gamma responses both below (Klopp et al., 1999, Lachaux et al., 2005, Fisch et al., 2009, Engell and McCarthy, 2010) and above 50 Hz in response to static facial photographs (Vidal et al., 2010, Davidesco et al., 2014) and face-like stimuli (Lachaux et al., 2005). Most studies of this high-frequency gamma response may have employed intra-cranial measurements, instead of scalp EEG or MEG, because direct cortical contact facilitates resolution of the low-amplitudes associated with very high frequencies (Uhlhaas et al., 2011). Our data, which differs from this work by using MEG and dynamic facial video, do not show a compelling response amplitude component between 60 and 100 Hz (Fig. 4) and our exploratory RSA of the 50–100 Hz range did yield significant effects.

### Source reconstruction

We tested our primary hypotheses about similarity-space representations in the brain using MEG data in sensor space, as this is relatively close to the original signal. This sensor space analysis was framed as a search through frequency space for RSA effects. We supplemented this sensor-level analysis by isolating the evoked response and the frequency ranges at which sensor space RSA effects were best observed (<20 Hz, see Fig. 6) and testing for sources that express RSA effects associated with this frequency band. Consequently, this source space analysis was not aimed at establishing the frequencies at which evoked and induced signals showed similarity-based effects. Instead, we designed the source reconstructions analysis to suggest some sources from within the well-known occipitotemporal network of brain areas involved in perception of dynamic faces that might be responsible for the effects that we observed in sensor space. The network of brain areas responsible for high-level representation of form and motion in dynamic faces is not controversial, can be observed in individual participants, as well as across participant samples and has been demonstrated many times using fMRI (Fox et al., 2009; Schultz and Pilz, 2009; Trautmann et al., 2009; Pitcher et al., 2011; Foley et al., 2012; Grosbras et al., 2012; Schultz et al., 2013; Furl et al., 2014, Furl et al., 2015), including within the present study, and using MEG (Furl et al., 2010, Sato et al., 2015). This network includes face-selective areas in bilateral FFA and OFA, motion-sensitive areas in bilateral V5 and an area responding preferentially to dynamic faces in right STS. A homologous pattern of areas that are face-selective, motion-sensitive and specific to dynamic faces has been observed in the macaque (Furl et al., 2012). Given our a priori expectation of involvement of these areas, and the need for informative priors to facilitate accurate source reconstruction, we constrained our source reconstructions to this network. We measured responses from source locations obtained from fMRI localization using the same participants as our MEG sample. We then tested whether different areas within this network, individually, exhibited our hypothesized similarity spaces. This finding is not guaranteed. The response patterns with the requisite similarity may be distributed over the network and response patterns over the neural population within any one region may not individually provide sufficient information. At the same time, it must be remembered that these areas are closely-spaced within occipitotemporal cortex, relative to the potential spatial resolution of EBB source reconstruction. Thus, the source reconstruction results should be interpreted with caution. Nevertheless, our results did implicate specific foci within the network as potential sources for our evoked and oscillatory RSA effects: bilateral FFA and V5. The involvement of V5 in representing some types of information, such as configural form, seems surprising, given that V5 was defined as motion-sensitive. Although, there is evidence that V5 can contribute to perception of static images, when only form is available (Furl et al., 2012), this effect could also arise from imprecise source resolution. The relationship of FFA with identity and form is consistent with prevailing views of the function of FFA (Haxby et al., 2001; Calder et al., 2005; Bernstein and Yovel, 2015), although our data suggest a wider role, including motion as well.

## Conclusion

We show that oscillations convey information about dynamic faces that evoked responses do not. Specifically, they support a broad, general encoding that includes both physical information about static facial form configurations and how these movements are patterned in time. This oscillatory coding, moreover, correlates with behavioral measures of facial form and motion perception. And, this oscillatory encoding captures the similarity structure associated with identity, even though there is further form and motion information that is not related to identity also. These results are consistent with a hypothesis that these oscillations may reflect the multidimensional basis for a “face space” – a popular and longstanding theoretical viewpoint in face perception – in which faces are represented by their similarity on multiple attribute dimensions (e.g., form, motion).
